# Emergent Anesthetic Management in a Patient Recently Diagnosed With Stiff Person Syndrome

**DOI:** 10.7759/cureus.70899

**Published:** 2024-10-05

**Authors:** Ana Sofia Pinto, Rita Lopes Dinis, Pedro Godinho, Cristina Carmona

**Affiliations:** 1 Department of Anesthesiology and Pain Therapy, Hospital Professor Doutor Fernando Fonseca, Amadora, PRT

**Keywords:** emergent splenectomy, gamma-aminobutyric acid (gaba), general anesthesia, rare disease, stiff person syndrome, total intravenous anesthesia (tiva)

## Abstract

Stiff person syndrome (SPS) is a rare autoimmune neurological disorder characterized by muscle rigidity and episodic spasms that involve axial and limb musculature. It has important implications during anesthesia, as it leads to gamma-aminobutyric acid (GABA)-mediated inhibitory networks malfunction. This report describes the anesthetic management of a 56-year-old patient with SPS and hereditary spherocytosis undergoing emergent splenectomy due to splenic hematoma and hemoperitoneum after a fall. Total intravenous anesthesia (TIVA) was performed with the adjunctive administration of rocuronium in order to obtain adequate intubation and surgical conditions. Careful management of patients with SPS is strongly suggested given their sensitivity to inhalational anesthetics and neuromuscular blockers, which can lead to hypotonia and muscle weakness requiring maintenance of mechanical ventilation in the postoperative period.

## Introduction

Stiff person syndrome (SPS) is a rare neurological condition first described in 1956 by Moersch and Woltman, with an estimated incidence of 1:1.000.000 [1]. Clinical symptoms typically manifest in the fourth decade of life and are characterized by muscle rigidity of the axial and limb musculature due to continuous co-contracture of the agonist and antagonist muscles; sudden painful spasms triggered by unexpected noises, tactile or visual stimuli, pain, and emotional stress [1,2].

SPS is also frequently associated with other autoimmune diseases, such as diabetes mellitus, which affects up to 35% of the patients, thyroiditis, and pernicious anemia [2]. The underlying pathophysiology of SPS is related to autoantibodies that attack the glutamic acid decarboxylase (GAD) enzyme, which is crucial for the synthesis of GABA. Although a positive serology is not required for the diagnosis (about 60% of patients have anti-GAD antibodies in the blood and cerebrospinal fluid (CSF)), reduced GABA production leads to decreased inhibitory signals in various brain regions, including the cortex, striated nucleus, and basal ganglia. This results in chronic motor neuron activation, causing muscle spasms and rigidity [3,4]. The management of SPS typically includes GABA-enhancing drugs (such as sedative anxiolytics and antiepileptic drugs), antispasticity drugs (such as baclofen, tizanidine, and dantrolene), and immunomodulating therapies. Benzodiazepines, like diazepam, are considered the first-line treatment as they enhance GABA_A_ receptor opening [2].

A notable concern in SPS management is the interaction of anesthetic drugs with GABA receptors. Clinical reports have documented hypotonia, delayed awakening, and prolonged mechanical ventilation following general anesthesia with muscle relaxants and volatile anesthetics, especially in those receiving baclofen, a GABA_B_ agonist agent [2,5]. This case report describes the anesthetic management of a patient with SPS who required emergency splenectomy under general anesthesia due to hemoperitoneum following a fall.

## Case presentation

A 56-year-old male (height 176 cm, weight 92 kg) with a medical history of hereditary spherocytosis was admitted to the neurology department for the investigation of muscle weakness in the lower limbs, accompanied by pronounced stiffness and generalized myotatic hyperreflexia, of a six-week duration. The patient had previously presented to the emergency department, where he was discharged with a prescription for non-steroidal anti-inflammatory drugs (NSAIDs), but his clinical condition deteriorated, and he experienced a fall and progressive loss of mobility.

Neurological evaluation revealed hypertonia with spasticity in all four limbs, generalized myotatic hyperreflexia, and slight weakness in the left lower limb (grade four out of five). The patient also reported painful spasms in the lower limbs during hospitalization. The etiological investigation revealed reactive hematopoietic bone marrow hyperplasia and marked homogeneous splenomegaly (measuring 18.3 cm x 20 cm), consistent with the patient's known medical records. Both the electromyogram and CSF analysis were normal. The patient was initiated on a treatment regimen of clonazepam, methylprednisolone, and baclofen, resulting in improvements in spasticity, painful spasms, and the ability to walk by the third day of treatment.

**Figure 1:**
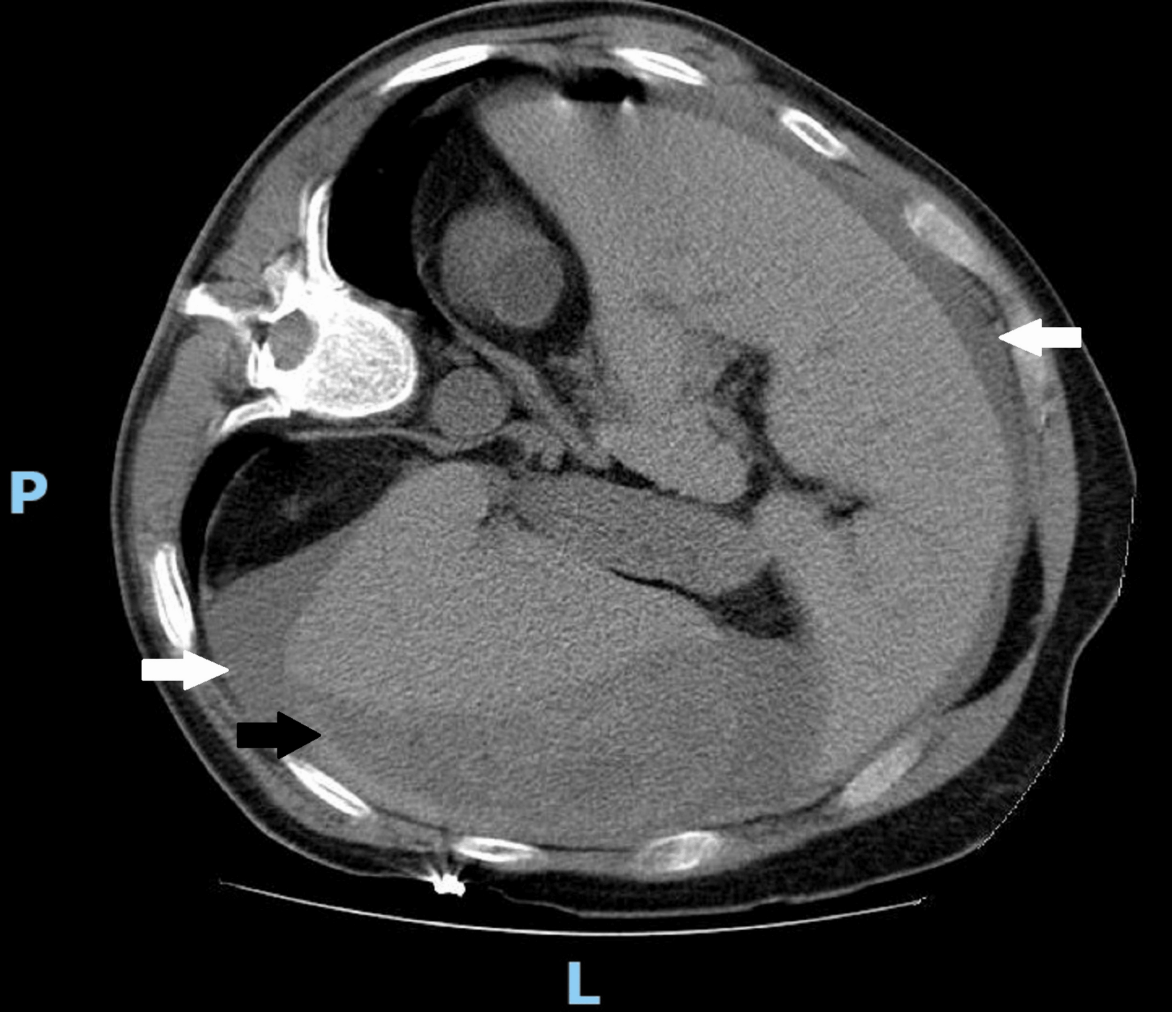
Abdominal computed tomography showing large hemoperitoneum (white arrows) and splenic subcapsular hematoma (black arrow) L: left; P: posterior

On the seventh day of hospitalization, following a fall from a standing height, a splenic subcapsular hematoma with hemoperitoneum was documented (Figure 1), and an emergency laparotomy with splenectomy was performed. Previous medical history included ophthalmic and orthopedic surgery before the SPS diagnosis, without recorded complications.

**Figure 2:**
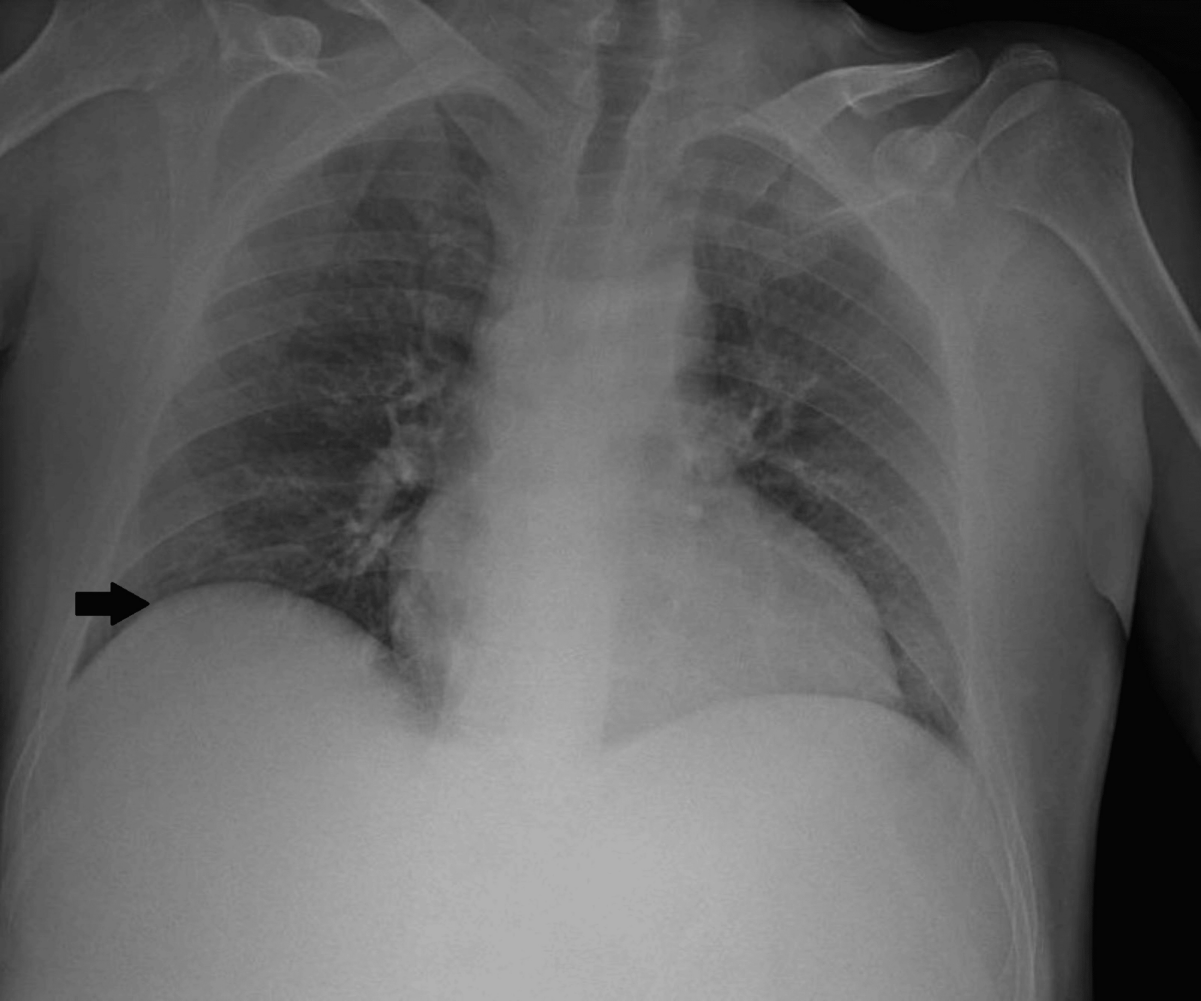
Chest X-ray with a slight elevation of the right hemidiaphragm (black arrow) and an enlarged cardiothoracic ratio

Regarding the preoperative tests, the patient showed a decrease in hemoglobin from 9.8 to 8.9 g/dL and worsening renal function in a probable prerenal context, with an increase in creatinine from 0.82 to 2.25 mg/dL. The chest X-ray (Figure 2), the remaining laboratory results, as well as the electrocardiogram, showed no significant abnormalities in this context.

On admission to the operating room, the patient was obnubilated, with slightly low blood pressure (98/58 mmHg) and sinus tachycardia (114 bpm). He was monitored according to standard American Society of Anesthesiologists (ASA) guidelines, with additional monitoring of invasive blood pressure, neuromuscular blockade (TOFscan® (Dräger, Lübeck, Germany), which monitors the adductor pollicis muscle), and depth of anesthesia (BIS^TM^ monitoring system (Medtronic plc, Minneapolis, MN, USA)). Anesthetic induction was performed with propofol (80 mg), ketamine (50 mg), fentanyl (0.15 mg), and rocuronium (80 mg). The patient did not exhibit signs of a difficult airway nor any limitations in cervical extension so airway management was performed under deep anesthetic conditions using direct laryngoscopy. The procedure lasted two and a half hours, during which anesthesia was maintained with a target-controlled infusion of propofol. The patient was warmed with a forced air warming blanket and heated intravenous fluids, and analgesia was administered with fentanyl (0.05 mg), paracetamol (1000 mg), and metamizole (2000 mg). Resuscitation was conducted with 2000 mL of crystalloids, 3000 mg of fibrinogen, 1000 mg of tranexamic acid, three units of red blood cell concentrate, and three units of fresh frozen plasma, addressing an estimated blood loss of 3100 mL. Urinary output during the surgical procedure was 0.65 mL/kg/h. The patient remained hemodynamically stable throughout the surgery and there was no need to administer vasoactive drugs. At the end of the procedure, a morphine infusion was initiated at 2 mg/h following a 6 mg bolus. Based on the TOFscan® monitoring system, the patient was successfully extubated after administering 200 mg of sugammadex and transferred to the intensive care unit, where he remained for three days without complications.

The patient was subsequently transferred to the Neurology ward and was discharged on the eighteenth day of hospitalization with significant improvement in rigidity, particularly in the upper limbs, while maintaining the ability to walk independently. He continued treatment with baclofen and clonazepam and underwent six cycles of intravenous immunoglobulin in the outpatient clinic, resulting in a nearly complete resolution of symptoms.

## Discussion

Due to the extremely low incidence of the disease, limited data is available on the anesthetic management of patients with SPS. However, considering the surgical procedure, total intravenous anesthesia (TIVA) appeared to be the most appropriate and widely accepted choice. Propofol is a safe choice for induction, and other good alternatives include thiopental [6]. These agents should reduce postoperative muscle spasms and pain in SPS patients. Ferrandis et al. have reported etomidate as having a higher risk of intraoperative myoclonus [7]. Propofol has also been implicated to cause intraoperative myoclonus, but it has a lower risk [8,9]. Moreover, it improves postoperative muscle rigidity and spasms, as reported by Obara et al. [10]. The published literature suggests that propofol reduces spinal activity through its agonistic action on GABA_A_ receptors, with some partial effect on central GABA_B_ receptors unlike baclofen, which is a selective GABA_B_ receptor agonist [11-13]. There is even a report of immediate improvement in acute symptoms following continuous propofol infusion [12]. It is known that volatile anesthetics cause muscular relaxation and potentiate the effects of non-depolarizing neuromuscular blockers through their inhibitory actions on GABA_B_ receptors, and hypotonia and prolonged weakness after general anesthesia with these agents have been reported. The proposed hypothesis is that there may be an interaction between volatile agents and baclofen. So, baclofen amplifies the gabanergic effects of volatile agents during general anesthesia by extending postsynaptic inhibitory currents when GABA is released [5]. Therefore, the dose of volatile anesthetics should be adjusted in patients undergoing treatment with baclofen [6]. The use of ketamine in SPS patients is rarely reported in the literature. Hanna et al., in a recent case report, described the use of monthly ketamine infusions to alleviate symptoms of pain and muscle spasms [14]. Currently, ketamine is utilized in various neurological and autoimmune diseases for symptom management. However, its use in the context of anesthetic induction, as described in our case, is scarcely reported [14]. Based on current evidence, propofol seems to be the drug of choice for anesthesia induction in SPS patients, with concomitant use of midazolam, which also modulates GABA_A_ receptor activity [6]. Since the patient was already managed with benzodiazepine and baclofen, and given the emergency context with an altered state of consciousness, we decided not to administer premedication.

There is conflicting evidence on the use of muscle relaxants [7,15]. Baclofen is a centrally acting muscle relaxant that suppresses monosynaptic and polysynaptic reflex transmission in the spinal cord by stimulating GABA_B_ receptors without affecting neuromuscular transmission. Peripheral muscle relaxants, on the other hand, act on the postsynaptic nicotinic acetylcholine receptors at the motor endplate, either causing depolarization or binding to the receptor, resulting in competitive blockade [3]. Johnson and Miller reported cases of muscular weakness following general anesthesia with muscle relaxants despite appropriate reversal, and the need for postoperative mechanical ventilation for up to 48 hours is also reported in the literature [15]. However, Obara et al. reported that the use of neuromuscular blockers was not associated with a greater or more prolonged response than usual [5,10]. It seems that there is no anesthetic interaction or additional contraindication to use neuromuscular blocking agents in SPS patients [6,16], and it is possible that the prolonged weakness reported in some cases could be due to an interaction between the volatile agents and GABA receptors or from the volatile agents and other commonly used medications, such as baclofen or diazepam, as reported by Bouw et al. and Johnson and Miller [5,15]. Careful individual monitoring of neuromuscular response in the form of TOF is important and rocuronium/vecuronium would be the recommended drugs of choice, which can be reversed promptly with sugammadex even in situations of a profound neuromuscular blockade [17].

In our case report, we decided to use rocuronium as it was an emergent procedure, to improve intubation conditions (since muscle rigidity can impair optimal intubation position) and to provide better surgical conditions. There were no complications during anesthetic recovery, and neuromuscular blockade reversal occurred as expected, without any signs of associated muscle weakness at the time of extubation. Since there was no need to administer additional doses of rocuronium, it could be speculated that a prolonged action of this drug may have occurred in this case, and this may be taken into consideration when planning the anesthetic plan of these patients. Given that the patient was on baclofen therapy, maintaining an appropriate temperature was also a concern, especially considering the administration of a substantial amount of fluids and blood products during surgery. Patients under baclofen usually have poor temperature perception, as it increases both the threshold for warm and cold stimuli through the activation of GABA_B_ receptors [6,18]. Although it did not apply to this clinical case, both neuraxial regional anesthesia and local anesthesia appear to be safe options, although spine deformity and rigidity may pose technical challenges. Additionally, it is important to note that pain or discomfort may trigger painful spasms [19,20].

Finally, it is recommended that postoperative surveillance occur in a level II or III care unit due to the increased risk of hypotonia and respiratory depression. Although the patient did not exhibit significant organ dysfunction that would warrant admission to an intensive care unit, the multidisciplinary team considered that the postoperative period should be managed at this level of care due to the patient’s recent diagnosis of a rare syndrome, as well as the surgical complexity and associated blood loss.

## Conclusions

SPS is a rare disease with a suspected autoimmune etiology that can present anesthetic challenges due to its effects on the GABA pathway. The use of a TIVA with propofol and opioids is a suitable option. Although some reports associate volatile anesthetics with muscle weakness, they appear to be safe, as does rocuronium, which can be reversed with sugammadex if necessary.

Explaining the procedure and the use of premedication with a benzodiazepine can relax the patient and prevent any spasms or other SPS symptomatology. The patient's anesthetic depth and neuromuscular blockade should be under continuous monitoring. Given the low incidence of this disease, more data are needed for the optimal anesthetic approach of these patients.
